# The optimal pre-post allocation for randomized clinical trials

**DOI:** 10.1186/s12874-023-01893-w

**Published:** 2023-03-28

**Authors:** Shiyang Ma, Tianying Wang

**Affiliations:** 1 Clinical Research Institute, Shanghai Jiao Tong University School of Medicine, Shanghai, China; 2 Clinical Research Center, Renji Hospital, Shanghai Jiaotong University School of Medicine, Shanghai, China; 3 Center for Statistical Science, Tsinghua University, Beijing, China; 4 Department of Industrial Engineering, Tsinghua University, Beijing, China

**Keywords:** Optimal allocation, Repeating baselines, Pre-post design, Analysis of covariance, Repeated measures

## Abstract

**Background:**

In pre-post designs, analysis of covariance (ANCOVA) is a standard technique to detect the treatment effect with a continuous variable measured at baseline and follow-up. For measurements subject to a high degree of variability, it may be advisable to repeat the pre-treatment and/or follow-up assessments. In general, repeating the follow-up measurements is more advantageous than repeating the pre-treatment measurements, while the latter can still be valuable and improve efficiency in clinical trials.

**Methods:**

In this article, we report investigations of using multiple pre-treatment and post-treatment measurements in randomized clinical trials. We consider the sample size formula for ANCOVA under general correlation structures with the pre-treatment mean included as the covariate and the mean follow-up value included as the response. We propose an optimal experimental design of multiple pre-post allocations under a specified constraint, that is, given the total number of pre-post treatment visits. The optimal number of the pre-treatment measurements is derived. For non-linear models, closed-form formulas for sample size/power calculations are generally unavailable, but we conduct Monte Carlo simulation studies instead.

**Results:**

Theoretical formulas and simulation studies show the benefits of repeating the pre-treatment measurements in pre-post randomized studies. The optimal pre-post allocation derived from the ANCOVA extends well to binary measurements in simulation studies, using logistic regression and generalized estimating equations (GEE).

**Conclusions:**

Repeating baselines and follow-up assessments is a valuable and efficient technique in pre-post design. The proposed optimal pre-post allocation designs can minimize the sample size, i.e., achieve maximum power.

**Supplementary Information:**

The online version contains supplementary material available at 10.1186/s12874-023-01893-w.

## Background

It is common in randomized clinical trials to collect information from patients before they enter the study. Typically eligibility for the trial is assessed at a screening visit, and a subsequent baseline visit is conducted prior to randomization to document clinical status at that time. Huntington disease studies for tetrabenazine and deutetrabenazine are randomized, placebo-controlled clinical trials (Huntington Study Group [1, 2]). As a motivation of this paper, the primary measure for both Huntington disease studies was the total chorea score of the Unified Huntington’s Disease Rating Scale, analyzed as a continuous variable. The total chorea score was measured at screening, baseline, and several follow-up visits. The treatment effect was evaluated using analysis of covariance (ANCOVA) model. In ANCOVA, both studies used the average baseline scores (i.e., the average values of two pre-treatment measurements made at screening and at true baseline) as the covariate and the change from baseline as the dependent variable. The question then arises, “What are the benefits of using multiple pre-treatment measurements?”

The use of multiple pre-treatment measurements in randomized clinical trials has been proposed in recent years. In a randomized controlled trial for the effect of soy phytoestrogens on hot flashes in women with breast cancer, the hot flash scores were measured every 24 hours for 4 weeks baselines and 12 weeks follow-ups [3]. A variety of endpoints, such as daily scores of migraine headache and brief fatigue inventory, were also assessed at multiple pre-treatment and post-treatment measurements [4]. Besides, several statistical papers discuss repeating the pre-treatment measurements for pre-post design. Frison and Pocock [5] demonstrated the merits of using more than one pre-treatment measurement in ANCOVA, with the pre-treatment mean as the covariate and the post-treatment mean as the outcome. Bristol [6] presented simulation studies using two pre-treatment measurements as covariates in linear regression models. Zhang et al. [7] considered the power analysis of choosing two baselines in ANCOVA for continuous variables and in logistic regression for categorical variables by simulation studies.

ANCOVA is a common technique to incorporate the baseline value as the covariate and estimate the treatment effect in randomized clinical trials. Standard theory, based on linear regression models, shows that the adjustment for a covariate reduces the residual variance by a factor of $1- \rho ^2$, where $\rho$ is the correlation between the covariate and the outcome [8]. That would increase the precision of detecting the treatment effect. Alternative approaches treat the pre-treatment measurements as additional outcome variables in mixed effects analysis. This was exemplified by Liang and Zeger [9] and Tango [10]. These authors showed that the generalized linear mixed-effects model is another efficient tool for pre-post design, which could extend to discrete responses with non-linear models.

In randomized clinical trials with repeated measures, investigators usually focus on repeating the follow-up assessments, which is generally more advantageous than repeating the pre-treatment measurements. However, the latter can still be valuable and was ignored by most of the clinical trials. In this paper, we address the benefits of repeating the baselines using the ANCOVA model, which would be an interesting and novel point of randomized controlled clinical trials. Besides, when there are multiple pre-treatment and post-treatment measurements, we investigate the optimal pre-post allocation to minimize the required sample size. In the section Methods, we consider the ANCOVA sample size formula using multiple pre-post measurements under a general unequal correlation structure. We further derive the optimal number of pre-treatment and post-treatment measurements given the total number of pre-post visits. In section Results, we illustrate the above procedures using the “Beat the Blues” data from a clinical trial of an interactive multimedia program [11]. In simulation studies, we consider both continuous and binary outcomes. When the outcome is binary, exact formulas are generally not available but simulation studies show that repeating baselines is advantageous under logistic regression., We use simulation studies to assess how well the formulas and insights from the ANCOVA case extend to binary outcomes. Merits and future works of the proposed optimal design are in the last two sections.

## Methods

### Repeating pre-treatment measurements in ANCOVA

We consider the ANCOVA model with the mean of multiple pre-treatment measurements as the covariate and the post-treatment mean as the outcome. Consider normally distributed endpoints in a randomized clinical trial and suppose that there are two treatment groups $i=0, 1$ (for placebo and treatment) with $n_i$ individuals per group. For all individuals, assume there are *S* pre-treatment visits and *T* post-treatment visits. Denote the pre-treatment measurements as $X_{ijs}$ and the post-treatment measurements as $Y_{ijt}$, where $i=0, 1,\ j=1,\ldots , n_{i}, s=1,\ldots , S$ and $t=1,\ldots , T$. We assume the $S+T$ pre-post measurements $(X_{ij1}, \ldots , X_{ijS}, Y_{ij1}, \ldots , Y_{ijT})^\prime$ follows multivariate normal distribution with mean $\varvec{\mu }=(\mu _{ij1}^{\text {pre}}, \ldots , \mu _{ijS}^{\text {pre}}, \mu _{ij1}^{\text {post}}, \ldots , \mu _{ijT}^{\text {post}})^\prime$ for $i=0 \ \text {or} \ 1$ and the $(S+T) \times (S+T)$ variance-covariance matrix
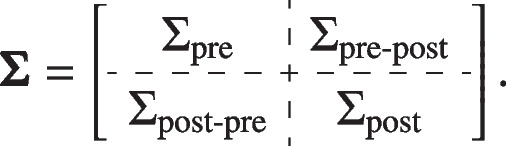


Denote the pre-treatment visits mean as $\bar{X}_{ij \cdot }= \sum _{s=1}^S X_{ijs} /S$ and the post-treatment visits mean as $\bar{Y}_{ij \cdot }= \sum _{t=1}^T Y_{ijt} /T, i=0, 1,\ j=1, \ldots , n_{i}$. The overall pre-treatment mean $\bar{X}=\sum _{i=0}^1 \sum _{j=1}^{n_i} \bar{X}_{ij \cdot } / (n_0+n_1)$. The ANCOVA model is

1 $$\begin{aligned} \bar{Y}_{ij \cdot }=\mu ^{\text {post}}_{i \cdot } + \beta (\bar{X}_{ij \cdot } - \bar{X}) + \epsilon _{ij}, \quad \epsilon _{ij} {\mathop {\sim }\limits ^{iid}} N(0, \sigma ^2). \end{aligned}$$

The estimated treatment effect $\hat{\delta }=\hat{\mu }^{\text {post}}_{1 \cdot }-\hat{\mu }^{\text {post}}_{0 \cdot }$, which is an unbiased estimator with variance formula [5, 12]:

$$\begin{aligned} \text {var}(\hat{\delta })= & {} \hat{\sigma }^2 \left[ \frac{1}{n_0}+\frac{1}{n_1}+\frac{(\bar{X}_{1 \cdot \cdot }-\bar{X}_{0 \cdot \cdot })^2}{\sum _{i=0}^1 \sum _{j=1}^{n_i} (\bar{X}_{ij \cdot }-\bar{X})^2} \right] \nonumber \\= & {} \frac{1}{n_0+n_1-3} \left[ \frac{1}{n_0}+\frac{1}{n_1}+\frac{(\bar{X}_{1 \cdot \cdot }-\bar{X}_{0 \cdot \cdot })^2}{\sum _{i=0}^1 \sum _{j=1}^{n_i} (\bar{X}_{ij \cdot }-\bar{X})^2} \right] \nonumber \\\times & {} \left\{ \sum _{i=0}^1 \sum _{j=1}^{n_i} (\bar{Y}_{ij \cdot }-\bar{Y}_{i \cdot \cdot })^2 - \frac{[\sum _{i=0}^1 \sum _{j=1}^{n_i}(\bar{X}_{ij \cdot }-\bar{X}_{i \cdot \cdot })(\bar{Y}_{ij \cdot }-\bar{Y}_{i \cdot \cdot })]^2}{\sum _{i=0}^1 \sum _{j=1}^{n_i}(\bar{X}_{ij \cdot }-\bar{X}_{i \cdot \cdot })^2}\right\} \nonumber \\= & {} \frac{n_0+n_1-2}{n_0+n_1-3} \left[ \frac{1}{n_0}+\frac{1}{n_1}+\frac{(\bar{X}_{1 \cdot \cdot }-\bar{X}_{0 \cdot \cdot })^2}{(n_0+n_1-2) \bar{\Sigma }_{\text {pre}} }\right] \bar{\Sigma }_{\text {post}} \left( 1- \frac{ \bar{\Sigma }_{\text {pre-post}}^2}{\bar{\Sigma }_{\text {pre}} \bar{\Sigma }_{\text {post}}} \right) \nonumber \\\approx & {} \left( \frac{1}{n_0}+\frac{1}{n_1} \right) \left( \bar{\Sigma }_{\text {post}}- \frac{ \bar{\Sigma }_{\text {pre-post}}^2}{\bar{\Sigma }_{\text {pre}}} \right) , \nonumber \end{aligned}$$

where $\bar{\Sigma }_{\text {pre}}, \bar{\Sigma }_{\text {post}}$ and $\bar{\Sigma }_{\text {pre-post}}$ are the mean of all elements in matrices $\Sigma _{\text {pre}}, \Sigma _{\text {post}}$ and $\Sigma _{\text {pre-post}}$, respectively. Term $(\bar{X}_{1 \cdot \cdot }-\bar{X}_{0 \cdot \cdot })^2$ can be negligible due to randomization and $(n_0+n_1-2) / (n_0+n_1-3)$ tends to 1 as sample size increases, which leads to the simple approximation [5].

Assume the covariance matrix
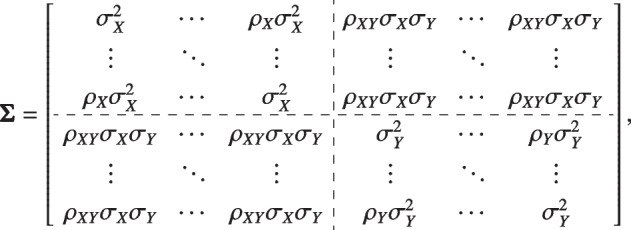


then we have $\bar{\Sigma }_{\text {pre}}=\sigma _X^2 [1+(S-1)\rho _X] /S, \bar{\Sigma }_{\text {post}}=\sigma _Y^2 [1+(T-1)\rho _Y] /T$ and $\bar{\Sigma }_{\text {pre-post}}=\rho _{XY} \sigma _X \sigma _Y$. The variance formula of ANCOVA becomes

2 $$\begin{aligned} \text {var}(\hat{\delta })\approx & {} \left( \frac{1}{n_0}+\frac{1}{n_1} \right) \sigma _Y^2 \left[ \frac{1+(T-1)\rho _Y}{T}-\frac{\rho _{XY}^2 S}{1+(S-1)\rho _X} \right] \nonumber \\= & {} \left( \frac{1}{n_0}+\frac{1}{n_1} \right) \sigma _Y^2 \left[ \frac{1-\rho _Y}{T}+\frac{(1-\rho _X) \rho _{XY}^2/\rho _X}{1+(S-1)\rho _X}+ \rho _Y -\frac{\rho _{XY}^2}{\rho _X} \right] . \end{aligned}$$

The merits of repeating the pre-treatment visits ($S \ge 2$) can be obtained directly from the variance formula (2). Keep the number of post-treatment visits *T* and other parameters fixed, the variance decreases as the number of pre-treatment visits *S* increases. Besides, when $\rho _{XY}$ and other parameters are fixed, the higher the correlation between the pre-treatment visits $\rho _X$, the less benefit is obtained by repeating the pre-treatment measurements. When $\rho _X$ is fixed, the higher the correlation between the pre- and post-randomization measurements $\rho _{XY}$, the variance becomes smaller, and the efficiency is gained from repeating pre-treatment visits.

The sample size formula per group under $n_0=n_1$ of *S* pre- and *T* post-treatment measurements is:

3 $$\begin{aligned} n(S,T) \approx \frac{2 \left[ \Phi ^{-1}(1-\alpha / 2)+\Phi ^{-1}(1-\beta ) \right] ^2 \sigma _Y^2 }{\delta ^2} \left[ \frac{1+ \rho _Y (T-1)}{T} - \frac{\rho _{XY}^2 S}{1+ \rho _X (S-1)} \right] , \end{aligned}$$

where $\delta$ is the treatment effect, $\alpha$ and $\beta$ are the Type I and Type II error probabilities levels. The merits of repeating the pre-treatment measurements can be obtained directly from $n(S=1,T=1)-n(S=2,T=1)\propto \frac{\rho _{XY}^2 (1-\rho _X)}{1+\rho _X}>0$.

As a simple numerical illustration, suppose that $\rho _X=\rho _Y=0.8, \rho _{XY}=0.6$, and the number of post-treatment visits $T=1$. The ratio of sample size formula (3) for having a single baseline visit ($S=1$) and having both screening and baseline visits ($S=2$) is $\frac{1+(T-1)\rho _Y-T\rho _{XY}^2}{1+(T-1)\rho _Y -2T\rho _{XY}^2/(1+\rho _X)}=1.067$. The omission of the second pre-treatment visit would lead to an increase in the sample size of 6.7%.

The same question may be asked about the benefit of repeating the post-treatment measurements. The ratio of sample sizes for using a single post-treatment measurement ($T=1$) and two post-treatment measurements ($T=2$) is $\frac{2 [1+\rho _X (S-1)] -2 \rho _{XY}^2 S }{(1+\rho _Y) [1+\rho _X (S-1)] -2 \rho _{XY}^2 S}$. Similarly, suppose $S=1$ and other parameters remain the same; this gives the ratio of sample sizes as 1.185. The omission of the second post-randomization evaluation would lead to an increase in the sample size of 18.5%. Hence, repeating the post-treatment measurements is more valuable than repeating the pre-treatment measurements in the ANCOVA model. The benefits combine if we repeat both pre-post measurements.

### Optimization of pre-treatment visits given the total number of visits

In this subsection, we address the related optimization problem when designing randomized clinical trials with multiple pre-post measurements. For a given total number of visits $M=S+T$, we are interested in the optimal number of pre-treatment visits $S_{\text {opt}}$, which minimizes the sample size.

First, we consider the equal correlation structure as $\rho _X=\rho _Y=\rho _{XY}=\rho$. Since $S+T=M$ is a fixed number and $\alpha , \beta , \delta , \sigma _Y^2, \rho$ are constant, minimizing the sample size $n \propto \frac{\rho (1-\rho ) M+ (1-\rho )^2}{(M-S) [1+ \rho (S-1)]}$is equivalent to maximizing the function $f(S)= (M-S) [1+ \rho (S-1)].$ This is a quadratic function with a negative leading coefficient under the assumption that $S \ge 1$. The optimal number of pre-treatment visits is

4 $$\begin{aligned} S_{\text {opt}}=\left\{ \begin{array}{ll} \frac{M}{2}-\frac{1-\rho }{2 \rho }, &{} \text {if}\ M\ge 1+ \frac{1}{\rho }, \\ 1, &{} \text {otherwise.}\\ \end{array} \right. \end{aligned}$$

Now we consider the sample size formula (3) under the unequal correlation structure. Minimizing the sample size formula is equivalent to minimizing the following objective function

$$\begin{aligned} f(S)= & {} \frac{1+ \rho _Y (M-S-1)}{M-S} - \frac{\rho _{XY}^{2} S}{1+ \rho _X (S-1)} \\= & {} \frac{[1+ \rho _X (S-1)] [1+ \rho _Y (M-S-1)] - \rho _{XY}^{2} S (M-S) }{(M-S) [1+ \rho _X (S-1)]} = \frac{P(S)}{Q(S)} \end{aligned}$$

for $1 \le S < M$. Notice this is a quotient of two quadratic polynomials of *S*.

#### Theorem 1

Assume $\rho _X \rho _Y- \rho _{XY}^2 \ge 0, 0< \rho _X, \rho _Y < 1$ and $\rho _{XY} \ne 0$. The objective function *f*(*S*) has a unique minimum point on $S \in [1, M)$ if $M \ge \sqrt{\frac{1-\rho _Y}{(1-\rho _X) \rho _{XY}^2}}+1$. The minimum point is

5 $$\begin{aligned} S_{\text {opt}}= \frac{ -(1- \rho _X) [M \rho _{XY}^2 + \rho _X (1-\rho _Y) ] + \rho _{XY} [1+ \rho _X (M-1)] \sqrt{(1-\rho _X) (1-\rho _Y)} }{ \rho _X^2 (1-\rho _Y) - \rho _{XY}^2 (1-\rho _X)}. \end{aligned}$$

Otherwise, if $M <\sqrt{\frac{1-\rho _Y}{(1-\rho _X) \rho _{XY}^2}}+1$, then $S_{\text {opt}}=1$.

#### Proof

The proof contains two parts: we first verify that the objective function *f*(*S*) has a unique minimum point on [1, *M*) and then derive the minimum point $S_{\text {opt}}$.

*Part 1: Uniqueness.* The two roots of the denominator *Q*(*S*) are $S=1-1/ \rho _X$ and $S=M$. Since $\rho _X>0$ and *Q*(*S*) has negative leading coefficient, $Q(S)>0$ for $S \in (1-1/ \rho _X, M)$. The numerator *P*(*S*) also has negative leading coefficient. Since $P (1-1/ \rho _X) =- \rho _{XY}^2 \left( 1-\frac{1}{\rho _X}\right) \left( M-1+\frac{1}{\rho _X} \right) > 0$ and $P(M)=[1+\rho _X (M-1)] (1-\rho _Y)> 0, P(S)>0$ for $S \in (1-1/ \rho _X, M)$. Therefore, $S=1-1/ \rho _X$ and $S=M$ are two vertical asymptotes of *f*(*S*), i.e., $\lim _{S \rightarrow (1-1/ \rho _X)^{+}} f(S)= +\infty$ and $\lim _{S \rightarrow M^{-}} f(S)= +\infty$.

Since $\rho _{XY} \ne 0, P(1-1/ \rho _X)>0$ and $P(M)>0, P(S)$ and *Q*(*S*) have no common zero. Equation $f(S)=P(S)/ Q(S)=k$ can be transformed into a quadratic equation, which has at most two roots. Hence, *f*(*S*) has a unique (relative) minimal point $s_0$ in $(1- 1/ \rho _X, M)$, which is absolute minimal point by our discussion. The function *f*(*S*) is decreasing in $( 1- 1/ \rho _X, s_0)$ and increasing in $(s_0, M)$. Therefore, if $s_0 \in [1,M), s_0$ is the minimal point; Otherwise, $S=1$ is the minimal point.

*Part 2: Derive* $S_{\text {opt}}$. The minimal point $s_0$ in $(1-1/ \rho _X, M)$ satisfies $f'(s_0)=0$. Obviously, the objective function can be written as

$$\begin{aligned} f(S)= \frac{A}{M-S} + \frac{B}{1+ \rho _X (S-1)}+ C, \end{aligned}$$

where $A=1-\rho _Y, B=\rho _{XY}^2 (1-\rho _X) /\rho _X$ and $C=\rho _Y-\rho _{XY}^2/\rho _X$. Then

$$\begin{aligned} f^\prime (S)= A /(M-S)^2 - B \rho _X /[1+ \rho _X (S-1)]^2. \end{aligned}$$

Since $A>0$ and $B \rho _X>0$, the only solution of $f^\prime (S)=0$ in $(1-1/ \rho _X, M)$ satisfies

$$\begin{aligned} \sqrt{A}/ (M-S)= \sqrt{B \rho _X}/ [1+ \rho _X (S-1)]. \end{aligned}$$

So

$$\begin{aligned} s_0=[M \sqrt{B \rho _X} - \sqrt{A} (1-\rho _X)]/ [\sqrt{B \rho _X} + \sqrt{A} \rho _X], \end{aligned}$$

which is

$$\begin{aligned} \frac{ -(1- \rho _X) [M \rho _{XY}^2 + \rho _X (1-\rho _Y) ] + \rho _{XY} [1+ \rho _X (M-1)] \sqrt{(1-\rho _X) (1-\rho _Y)} }{ \rho _X^2 (1-\rho _Y) - \rho _{XY}^2 (1-\rho _X)}. \end{aligned}$$

We can check that when $M \ge \sqrt{ \frac{1-\rho _Y}{(1-\rho _X) \rho _{XY}^2}}+1, s_0\ge 1$. So we have the conclusion. $\square$

#### Remark 1

When $\rho _{XY}=0$, the pre-treatment measures are unrelated to the post-treatment measures. Hence $S_{\text {opt}}=1$ under this special case. Also, since the $S_{\text {opt}}$ in (5) is usually not an integer, one should calculate the values of the objective function *f*(*S*) on both $\lfloor {S_{\text {opt}}}\rfloor$ and $\lceil {S_{\text {opt}}}\rceil$ and select the smaller one.

As an illustration, we assume that $\rho _{XY}=0.6, \rho _X=\rho _Y=0.8$, and the total number of visits $M=10$. Following Theorem 1, we obtain that $M=10> \sqrt{\frac{1-\rho _Y}{(1-\rho _X) \rho _{XY}^2}}+1=2.67$ and $S_{\text {opt}}=4.14$. Since $f(\lfloor {S_{\text {opt}}}\rfloor )=f(4)=0.4098 < f(\lceil {S_{\text {opt}}}\rceil )=f(5)=0.4114$, the optimal number of pre-treatment visits is $S=4$.

Now we consider a special case of $\rho _X=\rho _Y=\rho$ with the assumption $\rho \ge \rho _{XY}$. When $M \ge 1 / \rho _{XY}+1$,

$$\begin{aligned} S_{\text {opt}}= & {} \frac{ M \rho _{XY}^2 + \rho (1-\rho ) - \rho _{XY} (M \rho - \rho + 1)}{ \rho _{XY}^2- \rho ^2} =\frac{ M \rho _{XY} + \rho -1}{ \rho _{XY}+\rho } \left( = M \frac{\rho _{XY}}{\rho _{XY}+\rho }+\frac{\rho -1}{\rho _{XY}+\rho }< \frac{M}{2} \right) \\= & {} M- \frac{ (M-1)\rho +1}{ \rho _{XY}+\rho } = 1+ \frac{ (M-1)\rho _{XY} -1}{ \rho _{XY}+\rho }, \end{aligned}$$

which gives Eq. (4) under the further condition that $\rho _{XY}=\rho$. When fixing $\rho$, the higher the correlation between the pre-post measurements, the larger $S_{\text {opt}}$ is obtained. When fixing $\rho _{XY}$, the higher the correlation between two pre-treatment measurements or two post-treatment measurements, the smaller $S_{\text {opt}}$ is obtained.

In conclusion, when the total number of pre-post visits is fixed, one can obtain the optimal choice of *S* pre-treatment measurements and *T* post-treatment measurements to minimize the sample size. Measurements taken after the randomization can be more informative under the special case of $\rho _X=\rho _Y$ (since $S_{\text {opt}}<M/2$), while repeating the pre-treatment measurements is also valuable.

## Results

### Numerical example

We consider the “Beat the Blues” data from a clinical trial of an interactive multimedia program [11]. The data are available as the data frame “BtheB” in the R package HSAUR2. One hundred patients were allocated to the placebo group ($n_0=48$) and the treatment group ($n_1=52$). Each patient had $S=1$ baseline visit and $T=4$ post-treatment visits at 2, 3, 5, and 8 months after randomization.

Assume that these $S=1$ and $T=4$ measurements follow the unequal correlation structure with the variance-covariance matrix $\varvec{\Sigma }$. Based on the data set, we found that $\hat{\sigma }_X^2=117.5, \hat{\sigma }_Y^2=116.8, \hat{\rho }_{XY}=0.52$ and $\hat{\rho }_Y=0.77$. Since there is only $S=1$ pre-treatment visit, $\hat{\rho }_X$ could not be estimated. Instead, we simply assumed that $\hat{\rho }_X=\hat{\rho }_Y=0.77$. The treatment effect obtained from the dataset is $\hat{\delta }=5.4$. Using these estimates, we calculate the sample size per group (assume $n_0=n_1=n$) under $\alpha =0.05$ and $1-\beta =0.8$ using formula (3).

From Table 1, we verify that repeating the post-treatment measurements can be more valuable (with a smaller sample size) than repeating the pre-treatment measurements. The benefits combined if we repeat both pre-post measurements, e.g., $S=2, T=4$ can reduce up to 28.3% sample size compared with the single pre-post design ($S=1, T=1$). Note that in our numerical example, we consider a fixed power at 0.8 for different allocation strategies (See Table 1). The purpose of this example is to show that when power is fixed, more pre-treatment and post-treatment visits will lead to a smaller sample size per group, i.e., a more efficient trial. Equivalently, if the sample size is fixed, more *S* and *T* would lead to a more powerful analysis.

**Table 1:** Sample size per group *n*(*S*, *T*) for ANCOVA model under $\alpha =0.05$ and $1-\beta =0.8$ with different number of pre-treatment and post-treatment measurements

*S*, *T*	*n*(*S*, *T*)	Sample size reduction percentage (%) $1-n(S,T)/n(1,1)$
$S=1, T=1$	$n(1,1)=46$	-
$S=2, T=1$	$n(2,1)=44$	4.3%
$S=1, T=2$	$n(1,2)=39$	15.2%
$S=1, T=4$	$n(1,4)=36$	21.7%
$S=2, T=3$	$n(2,3)=35$	23.9%
$S=2, T=4$	$n(2,4)=33$	28.3%
$S=4, T=2$	$n(4,2)=36$	21.7%

We also derive the optimal number of pre-treatment visits *S* given the total number of visits $M=5$. Using formula (5) in Theorem 1, we obtain that $M \ge \sqrt{\frac{1-\rho _Y}{(1-\rho _X) \rho _{XY}^2}}+1=2.9$ and $S_{\text {opt}}=1.8$. Since for $\lfloor {S_{\text {opt}}}\rfloor =1, n(1,4)=36$ and for $\lceil {S_{\text {opt}}}\rceil =2, n(2,3)=35, S=2$ is the optimal number of pre-treatment visits. Hence, repeating the pre-treatment measurements ($S=2, T=3$) is superior to using a single baseline ($S=1, T=4$) under the constraint of the total number of visits $M=5$.

### Simulation studies

The previous algebra applies only to continuous measurements analyzed by the ANCOVA model. Other models are needed when the outcome variable is discrete. The exact formulas for power calculations are generally not available for non-linear models with binary outcomes. Hence, we set up Monte Carlo simulation studies to assess how well the formulas and insights from the ANCOVA model extend to the non-linear models. In this section, we conduct simulation studies on continuous and binary measurements. For continuous measurements, we use the ANCOVA model with the pre-treatment mean as covariate and the post-treatment mean as outcome. The binary outcomes are analyzed by logistic regression for a single outcome and by generalized estimating equations (GEE) for multiple outcomes. All simulation results were obtained using 20,000 replications.

#### Single / Multiple Continuous Outcomes

For a single continuous outcome, we assume there are $S=2$ and $T=1$ continuous measurements as $X_1$ (screening), $X_2$ (baseline), *Y* (outcome) and $(X_1, X_2, Y)$ follows MVN($\varvec{\mu }, \varvec{\Sigma }$). For the control group, $\varvec{\mu }=(0,0,0)$ and for treatment group, $\varvec{\mu }=(0,0,\delta )$. Assume $\sigma _X^2=\sigma _Y^2=1$. Different $\rho _{XY}$ and $\rho _X$ are considered: $\rho _{XY}=0.5, \rho _X=\{0.6, 0.7, 0.8, 0.9\}$; $\rho _{XY}=0.6, \rho _X=\{0.7, 0.8, 0.9\}$ and $\rho _{XY}=0.7, \rho _X=\{0.8, 0.9\}$. The sample sizes of the control and treatment groups are $n_0=n_1=\{50, 75, 100, 125, 150\}$.

The ANCOVA model (1) is considered of using only baseline ($S =1$) as the covariate or taking the mean of screening and baseline ($S=2$) as the covariate for a single continuous outcome *Y*. We set the effect size $\delta =0$ to evaluate Type I error probabilities and $\delta =0.3$ for power. The Type I error probabilities of ANCOVA models control well by using only baseline ($S=1$) or screening and baseline ($S=2$) (Table 2). The power of repeating pre-treatment measurements consistently exceeds the power of using a single baseline (Table 3). For $S=2$, when $\rho _{XY}$ is fixed, higher $\rho _{X}$ leads to lower power. When $\rho _X$ is fixed, higher $\rho _{XY}$ would obtain larger power.

**Table 2:** Type I error probabilities of using only baseline ($S=1$) or screening and baseline (S=2) for a single continuous outcome, under different sample sizes, $\rho _{XY}$ and $\rho _X$

	$S=1, T=1$	$S=2, T=1$	$S=1, T=1$	$S=2, T=1$
	$n_0=n_1=50$		$n_0=n_1=75$	
$\rho _{XY}=0.5, \rho _X=0.6$	0.0504	0.0494	0.0488	0.048
$\rho _{XY}=0.5, \rho _X=0.7$	0.0491	0.0495	0.049	0.0484
$\rho _{XY}=0.5, \rho _X=0.8$	0.0493	0.0494	0.0488	0.0483
$\rho _{XY}=0.5, \rho _X=0.9$	0.0494	0.0498	0.0491	0.0486
$\rho _{XY}=0.6, \rho _X=0.7$	0.0497	0.0495	0.0497	0.0488
$\rho _{XY}=0.6, \rho _X=0.8$	0.0492	0.0495	0.0503	0.0486
$\rho _{XY}=0.6, \rho _X=0.9$	0.0497	0.0494	0.0488	0.0482
$\rho _{XY}=0.7, \rho _X=0.8$	0.0491	0.0499	0.0491	0.0486
$\rho _{XY}=0.7, \rho _X=0.9$	0.0495	0.0498	0.0495	0.0488
	$n_0=n_1=100$		$n_0=n_1=125$	
$\rho _{XY}=0.5, \rho _X=0.6$	0.0485	0.0482	0.049	0.0488
$\rho _{XY}=0.5, \rho _X=0.7$	0.0497	0.048	0.0488	0.0489
$\rho _{XY}=0.5, \rho _X=0.8$	0.0498	0.048	0.0488	0.049
$\rho _{XY}=0.5, \rho _X=0.9$	0.049	0.0484	0.0496	0.0491
$\rho _{XY}=0.6, \rho _X=0.7$	0.0496	0.0484	0.0492	0.0495
$\rho _{XY}=0.6, \rho _X=0.8$	0.0502	0.0482	0.0485	0.0493
$\rho _{XY}=0.6, \rho _X=0.9$	0.0498	0.0484	0.0486	0.0493
$\rho _{XY}=0.7, \rho _X=0.8$	0.0498	0.0484	0.0493	0.05
$\rho _{XY}=0.7, \rho _X=0.9$	0.0506	0.0482	0.0491	0.0496
	$$n_0=n_1=150$$			
$\rho _{XY}=0.5, \rho _X=0.6$	0.05	0.0496		
$\rho _{XY}=0.5, \rho _X=0.7$	0.0498	0.0497		
$\rho _{XY}=0.5, \rho _X=0.8$	0.0488	0.0499		
$\rho _{XY}=0.5, \rho _X=0.9$	0.0491	0.0499		
$\rho _{XY}=0.6, \rho _X=0.7$	0.0502	0.0497		
$\rho _{XY}=0.6, \rho _X=0.8$	0.0495	0.0498		
$\rho _{XY}=0.6, \rho _X=0.9$	0.0484	0.0498		
$\rho _{XY}=0.7, \rho _X=0.8$	0.0505	0.0493		
$\rho _{XY}=0.7, \rho _X=0.9$	0.0494	0.0495		

**Table 3:** Power of using only baseline ($S=1$) or screening and baseline (S=2) for a single continuous outcome, under different sample sizes, $\rho _{XY}$ and $\rho _X$

	$S=1, T=1$	$S=2, T=1$	$S=1, T=1$	$S=2, T=1$
	$n_0=n_1=50$		$n_0=n_1=75$	
$\rho _{XY}=0.5, \rho _X=0.6$	0.4001	0.4324	0.554	0.5886
$\rho _{XY}=0.5, \rho _X=0.7$	0.4008	0.4225	0.554	0.5771
$\rho _{XY}=0.5, \rho _X=0.8$	0.4009	0.4132	0.5538	0.5674
$\rho _{XY}=0.5, \rho _X=0.9$	0.4019	0.4059	0.5532	0.5592
$\rho _{XY}=0.6, \rho _X=0.7$	0.456	0.4978	0.6206	0.6695
$\rho _{XY}=0.6, \rho _X=0.8$	0.4568	0.4819	0.6208	0.6526
$\rho _{XY}=0.6, \rho _X=0.9$	0.458	0.4692	0.6217	0.6354
$\rho _{XY}=0.7, \rho _X=0.8$	0.5428	0.5913	0.7212	0.766
$\rho _{XY}=0.7, \rho _X=0.9$	0.5446	0.5666	0.7194	0.7416
	$n_0=n_1=100$		$n_0=n_1=125$	
$\rho _{XY}=0.5, \rho _X=0.6$	0.6746	0.7116	0.775	0.8111
$\rho _{XY}=0.5, \rho _X=0.7$	0.6741	0.7	0.7736	0.7996
$\rho _{XY}=0.5, \rho _X=0.8$	0.6738	0.6908	0.7742	0.7907
$\rho _{XY}=0.5, \rho _X=0.9$	0.6748	0.6829	0.7737	0.782
$\rho _{XY}=0.6, \rho _X=0.7$	0.7428	0.7873	0.8381	0.8714
$\rho _{XY}=0.6, \rho _X=0.8$	0.7449	0.7706	0.8371	0.8586
$\rho _{XY}=0.6, \rho _X=0.9$	0.7446	0.7561	0.8371	0.8466
$\rho _{XY}=0.7, \rho _X=0.8$	0.8336	0.8744	0.9113	0.9378
$\rho _{XY}=0.7, \rho _X=0.9$	0.8334	0.8538	0.9113	0.9244
	$$n_0=n_1=150$$			
$\rho _{XY}=0.5, \rho _X=0.6$	0.8464	0.8766		
$\rho _{XY}=0.5, \rho _X=0.7$	0.8469	0.8686		
$\rho _{XY}=0.5, \rho _X=0.8$	0.8472	0.8603		
$\rho _{XY}=0.5, \rho _X=0.9$	0.8474	0.8534		
$\rho _{XY}=0.6, \rho _X=0.7$	0.8991	0.9266		
$\rho _{XY}=0.6, \rho _X=0.8$	0.899	0.9166		
$\rho _{XY}=0.6, \rho _X=0.9$	0.8989	0.9082		
$\rho _{XY}=0.7, \rho _X=0.8$	0.9507	0.9694		
$\rho _{XY}=0.7, \rho _X=0.9$	0.9507	0.9619		

For multiple continuous outcomes, we conduct simulation studies to obtain the optimal number of pre-treatment visits $S_{\text {opt}}$ given the total number of visits $M=10$. Similarly, we generate $M=10$ continuous measurements $(X_1, \ldots , X_S, Y_1, \ldots Y_T)$ using multivariate normal distribution with mean $\varvec{\mu }=(\mu _X, \ldots , \mu _X, \mu _Y, \ldots , \mu _Y)$ and covariance matrix $\varvec{\Sigma }$, where $S=\{1, \ldots , 9\}$ and $T=M-S$. For control group, $\mu _X=\mu _Y=0$ and for treatment group, $\mu _X=0, \mu _Y=\delta$. Again, assume $\sigma _X^2=\sigma _Y^2=1$. Different $\rho _{XY}$ and $\rho _X=\rho _Y$ are considered as above; $n_0=n_1=\{50, 100, 150\}$.

**Fig. 1:**
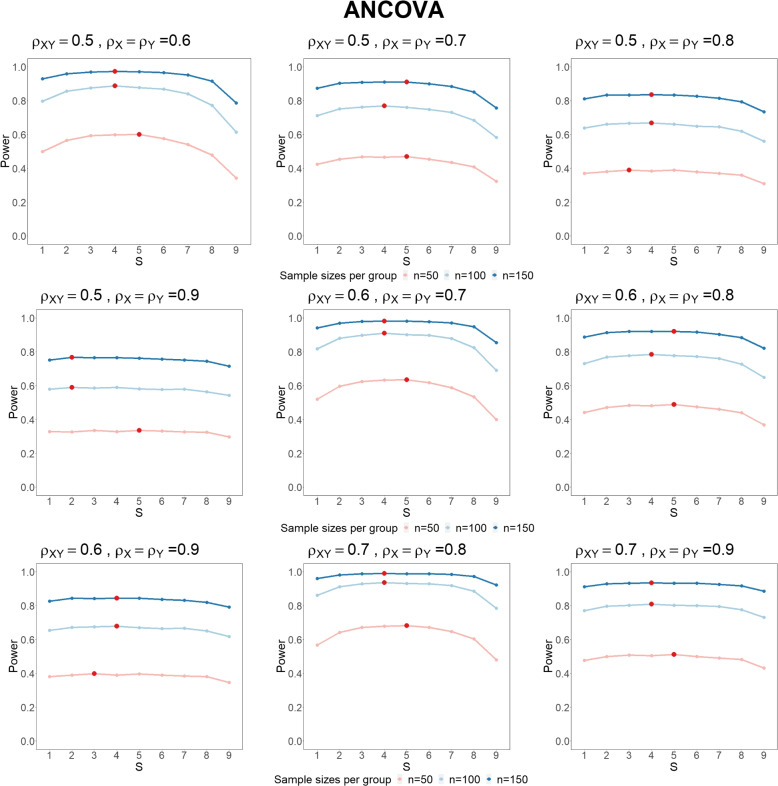
The power of multiple continuous outcomes using ANCOVA Model, with total number of visits $M=10$, sample size per group $n=n_0=n_1=\{50, 100, 150\}$ under different $\rho _{XY}, \rho _X$ and $\rho _Y$. The number of pre-treatment measurements $S=\{1, \ldots , 9\}$. The optimal number of pre-treatment visits $S_{\text {opt}}$ are highlighted in red points

We set the effect size $\delta =0$ to evaluate Type I error probabilities and $\delta =0.25$ for power. The Type I error probabilities all control well (Table S1). The power results (Fig. 1) show that having more than 2 pre-treatment visits can be more valuable than using a single baseline. The optimal number of pre-treatment visits is highlighted in red, showing that $S_{\text {opt}}$ are less than or equal to $M/2=5$. In summary, the simulation results give a similar conclusion as the ANCOVA analyses in the section Methods.

#### A Single Binary Outcome

Denote $S=2$ and $T=1$ binary measurements as $X_1, X_2$ and *Y*. We generate the correlated binary data using Gaussian copulas, which take the marginal of multivariate normal distributions to multivariate uniform distributions. Assume that the uniform margins $(U_{X_1}, U_{X_2}, U_{Y})$ has the correlation matrix

$$\begin{aligned} \varvec{R}= \left[ \begin{array}{ccc} 1 &{} \rho _X &{}\rho _{XY} \\ \rho _X &{} 1 &{}\rho _{XY} \\ \rho _{XY} &{} \rho _{XY} &{}1\\ \end{array} \right] . \end{aligned}$$

We then generate the Gaussian copulas under the correlation matrix $\varvec{R}$ using R package copula [13]. The correlated binary measurements are obtained below. For the control group, $(X_1, X_2, Y)= \left( 1_{(U_{X_1} \le p)}, 1_{(U_{X_2} \le p)}, 1_{(U_Y \le p)} \right)$. The dichotomized probability *p* yields triplets of dependent Bernoulli variables. For the treatment group, $(X_1, X_2, Y)= \left( 1_{(U_{X_1} \le p)}, 1_{(U_{X_2} \le p)}, 1_{(U_{Y} \le p^\prime )} \right)$, where $p^\prime =\frac{p e^{\beta _1}}{1-p+p e^{\beta _1}}, \beta _1$ represents the treatment effect coefficient, so that $\log \left( \frac{p^\prime }{1-p^\prime } \right) = \beta _1+ \log \left( \frac{p}{1-p} \right)$.

Three different logistic regression models are considered:

$$\begin{aligned} \text {Model 1 (only baseline)}: \quad \log \left( \frac{P(Y=1)}{1-P(Y=1)} \right)= & {} \beta _0+ \beta _1 \text {Treat}+ \beta _2 X_2, \\ \text {Model 2 (screening and baseline)}: \quad \log \left( \frac{P(Y=1)}{1-P(Y=1)} \right)= & {} \beta _0+ \beta _1 \text {Treat}+ \beta _2 X_{\text {log}},\\ \text {Model 3 (screening and baseline)}: \quad \log \left( \frac{P(Y=1)}{1-P(Y=1)} \right)= & {} \beta _0+ \beta _1 \text {Treat}+ \beta _2 X_C, \end{aligned}$$

where Treat is the treatment indicator, $X=X_1+X_2, X_C$ is categorial variable of *X* and $X_{\text {log}}=\text {log} \left[ (X+1/2)/(2-X+1/2) \right]$. The term 1/2 is introduced to avoid infinite estimates [14].

The logistic regression model

$$\begin{aligned} \log \left( \frac{P(Y=1)}{1-P(Y=1)} \right) = \beta _0+ \beta _1 \text {Treat}+ \beta _2 X \end{aligned}$$

is equivalent to Model 2 for $S=2$. That is because when $S=2, X=X_1+X_2=\{0,1,2\}$. Then $X_{\text {log}}=\text {log} [ (X+1/2)/ (2-X+1/2) ]= \{-\text {log}(5), 0, \text {log}(5)\}$, which is proportional to $X-1=\{-1,0,1\}$. Hence, using *X* or $X_{\text {log}}$ in the logistic regression model would provide exactly the same Type I error probabilities and power.

To detect the treatment effect, we consider the null hypothesis $H_0: \beta _1=0$ vs. the alternative hypothesis $H_1: \beta _1\ne 0$. Assume that the dichotomized probability $p=0.4$. The sample sizes of the control and treatment groups are $n_0=n_1=\{50, 75, 100, 125, 150\}$. Different $\rho _{XY}$ and $\rho _X$ (assume $\rho _{XY}<\rho _X$) are considered to generate the data, $\rho _{XY}=0.5, \rho _X=\{0.6, 0.7, 0.8, 0.9\}$; $\rho _{XY}=0.6, \rho _X=\{0.7, 0.8, 0.9\}$ and $\rho _{XY}=0.7, \rho _X=\{0.8, 0.9\}$. We conduct simulation studies with the treatment effect coefficient $\beta =0$ to obtain the Type I error probability and with $\beta =0.8$ to obtain power. For logistic regressions with small samples, perfect separation may occur, leading to infinite estimates of the logistic regression coefficient and fitted probabilities close to zero and one. Hence, when $n_0=n_1=50$, we only consider Models 1 and 2 in the simulation studies.

The simulation error for estimating the Type I error probability of $\alpha = 0.05$ is $1.96 \times \text {SE}=1.96 \times \sqrt{(0.05)(0.95)/20000}=0.003$. The Type I error probabilities of three different logistic regression models control well (See Table 4). Some of the Type I error probabilities are slightly conservative, which is reasonable for binary outcomes. The power results of three logistic regression models under different sample sizes, $\rho _{XY}$ and $\rho _X$ are shown in Table 5. The power of repeating pre-treatment measurements using $X_{\text {log}}$ or $X_C$ (Models 2, 3) consistently exceeds the power of using a single baseline $X_2$ (Model 1). When $\rho _{XY}$ is fixed, the higher the correlation between two pre-treatment measurements, the less benefit is obtained by repeating the pre-treatment measurements. When $\rho _X$ is fixed, the higher the correlation between the pre-post measurements, the larger power is obtained.

**Table 4:** Type I error probabilities of three different logistic regression models for a single binary outcome, under different sample sizes, $\rho _{XY}$ and $\rho _X$

	Model 1 ($X_2$)	Model 2 ($X_{\text {log}}$)	Model 3 $(X_{C})$	Model 1 ($X_2$)	Model 2 ($X_{\text {log}}$)	Model 3 $(X_{C})$
	$n_0=n_1=50$			$n_0=n_1=75$		
$\rho _{XY}=0.5, \rho _X=0.6$	0.0527	0.0517	-	0.05	0.0503	0.0512
$\rho _{XY}=0.5, \rho _X=0.7$	0.0526	0.052	-	0.0505	0.0502	0.0506
$\rho _{XY}=0.5, \rho _X=0.8$	0.0522	0.0519	-	0.0512	0.0504	0.052
$\rho _{XY}=0.5, \rho _X=0.9$	0.052	0.0526	-	0.0508	0.0505	0.0512
$\rho _{XY}=0.6, \rho _X=0.7$	0.051	0.0503	-	0.0508	0.0504	0.051
$\rho _{XY}=0.6, \rho _X=0.8$	0.0527	0.0517	-	0.0508	0.0503	0.0518
$\rho _{XY}=0.6, \rho _X=0.9$	0.0526	0.0511	-	0.0505	0.0494	0.05
$\rho _{XY}=0.7, \rho _X=0.8$	0.0508	0.0482	-	0.0506	0.0498	0.0508
$\rho _{XY}=0.7, \rho _X=0.9$	0.0499	0.049	-	0.051	0.0488	0.0493
	$n_0=n_1=100$			$n_0=n_1=125$		
$\rho _{XY}=0.5, \rho _X=0.6$	0.0479	0.0481	0.0491	0.0476	0.0476	0.0476
$\rho _{XY}=0.5, \rho _X=0.7$	0.0498	0.0491	0.0499	0.0494	0.0482	0.0494
$\rho _{XY}=0.5, \rho _X=0.8$	0.0488	0.0478	0.0493	0.0496	0.0491	0.05
$\rho _{XY}=0.5, \rho _X=0.9$	0.0482	0.0473	0.0484	0.0494	0.0504	0.0506
$\rho _{XY}=0.6, \rho _X=0.7$	0.0491	0.0472	0.0485	0.0476	0.0492	0.0503
$\rho _{XY}=0.6, \rho _X=0.8$	0.0485	0.0486	0.0492	0.0489	0.0486	0.0498
$\rho _{XY}=0.6, \rho _X=0.9$	0.0485	0.0493	0.0492	0.0471	0.0486	0.0487
$\rho _{XY}=0.7, \rho _X=0.8$	0.0469	0.0471	0.0482	0.0492	0.0497	0.0516
$\rho _{XY}=0.7, \rho _X=0.9$	0.0486	0.0472	0.0478	0.0483	0.049	0.05
	$$n_0=n_1=150$$					
$\rho _{XY}=0.5, \rho _X=0.6$	0.0486	0.0491	0.0493			
$\rho _{XY}=0.5, \rho _X=0.7$	0.049	0.0488	0.0488			
$\rho _{XY}=0.5, \rho _X=0.8$	0.0494	0.0493	0.0485			
$\rho _{XY}=0.5, \rho _X=0.9$	0.0488	0.048	0.0484			
$\rho _{XY}=0.6, \rho _X=0.7$	0.0495	0.0492	0.0499			
$\rho _{XY}=0.6, \rho _X=0.8$	0.0488	0.0499	0.0506			
$\rho _{XY}=0.6, \rho _X=0.9$	0.0489	0.0486	0.0494			
$\rho _{XY}=0.7, \rho _X=0.8$	0.0496	0.0504	0.0507			
$\rho _{XY}=0.7, \rho _X=0.9$	0.051	0.0504	0.051			

**Table 5:** Power of three different logistic regression models for a single binary outcome, under different sample sizes, $\rho _{XY}$ and $\rho _X$

	Model 1 ($X_2$)	Model 2 ($X_{\text {log}}$)	Model 3 $(X_{C})$	Model 1 ($X_2$)	Model 2 ($X_{\text {log}}$)	Model 3 $(X_{C})$
	$n_0=n_1=50$			$n_0=n_1=75$		
$\rho _{XY}=0.5, \rho _X=0.6$	0.5482	0.5662	-	0.7268	0.7509	0.749
$\rho _{XY}=0.5, \rho _X=0.7$	0.5474	0.5624	-	0.7264	0.7454	0.7444
$\rho _{XY}=0.5, \rho _X=0.8$	0.5476	0.5584	-	0.7262	0.7394	0.7405
$\rho _{XY}=0.5, \rho _X=0.9$	0.5464	0.555	-	0.7292	0.7361	0.7341
$\rho _{XY}=0.6, \rho _X=0.7$	0.5708	0.5946	-	0.754	0.7788	0.779
$\rho _{XY}=0.6, \rho _X=0.8$	0.5692	0.5896	-	0.7538	0.7732	0.771
$\rho _{XY}=0.6, \rho _X=0.9$	0.5668	0.5804	-	0.7558	0.767	0.7664
$\rho _{XY}=0.7, \rho _X=0.8$	0.6042	0.638	-	0.7888	0.819	0.818
$\rho _{XY}=0.7, \rho _X=0.9$	0.6028	0.6263	-	0.7889	0.808	0.8078
	$n_0=n_1=100$			$n_0=n_1=125$		
$\rho _{XY}=0.5, \rho _X=0.6$	0.8442	0.859	0.859	0.9134	0.9272	0.9261
$\rho _{XY}=0.5, \rho _X=0.7$	0.8426	0.8567	0.8558	0.9134	0.9244	0.9242
$\rho _{XY}=0.5, \rho _X=0.8$	0.8442	0.8536	0.8524	0.9134	0.9211	0.9213
$\rho _{XY}=0.5, \rho _X=0.9$	0.8418	0.8488	0.8489	0.9129	0.9182	0.9176
$\rho _{XY}=0.6, \rho _X=0.7$	0.8642	0.8864	0.8853	0.9288	0.9433	0.9428
$\rho _{XY}=0.6, \rho _X=0.8$	0.8644	0.8811	0.8804	0.9285	0.9396	0.9389
$\rho _{XY}=0.6, \rho _X=0.9$	0.8646	0.8765	0.875	0.9292	0.9354	0.9346
$\rho _{XY}=0.7, \rho _X=0.8$	0.8924	0.914	0.9139	0.9458	0.9598	0.9592
$\rho _{XY}=0.7, \rho _X=0.9$	0.892	0.9064	0.9058	0.9447	0.9552	0.954
	$$n_0=n_1=150$$					
$\rho _{XY}=0.5, \rho _X=0.6$	0.9536	0.9618	0.9608			
$\rho _{XY}=0.5, \rho _X=0.7$	0.9529	0.9598	0.9587			
$\rho _{XY}=0.5, \rho _X=0.8$	0.9522	0.9576	0.9571			
$\rho _{XY}=0.5, \rho _X=0.9$	0.9531	0.9554	0.955			
$\rho _{XY}=0.6, \rho _X=0.7$	0.9658	0.9732	0.9733			
$\rho _{XY}=0.6, \rho _X=0.8$	0.9643	0.9714	0.9711			
$\rho _{XY}=0.6, \rho _X=0.9$	0.963	0.9683	0.9679			
$\rho _{XY}=0.7, \rho _X=0.8$	0.9756	0.9834	0.9833			
$\rho _{XY}=0.7, \rho _X=0.9$	0.9748	0.9815	0.9808			

Hence, repeating the pre-treatment measurements is valuable under logistic regressions for a single binary outcome. This conclusion is the same as the ANCOVA model for continuous outcome variables, which shows that repeating the pre-treatment measurements have a nice performance extending to the binary variables using logistic regression.

#### Multiple Binary Outcomes

We conduct simulation studies to obtain the optimal number of pre-treatment visits $S_{\text {opt}}$ given the total number of visits $M=10$ under binary data. We use GEE logistic regression models [15] for correlated binary data when the number of post-treatment visits *T* exceeds one (multiple binary outcomes).

Similarly, we generate $M=10$ correlated binary measurements $(X_1, \ldots , X_S, Y_1, \ldots Y_T)$ using Gaussian copulas, where $S=\{1, \ldots , 9\}$ and $T=M-S$. The uniform margins $(U_{X_1}, \ldots , U_{X_S}, U_{Y_1}, \ldots , U_{Y_T})$ has a correlation matrix:
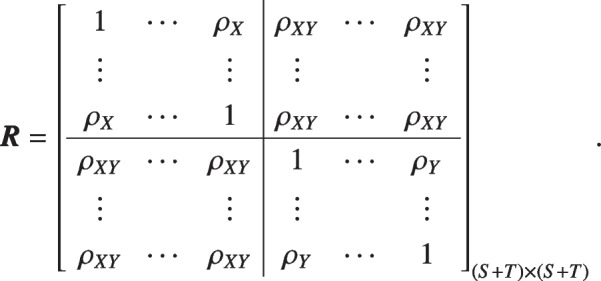


For the control group,$(X_1, \ldots , X_S, Y_1, \ldots , Y_T)= \left( 1_{(U_{X_1} \le p)}, \ldots , 1_{(U_{X_S} \le p)}, 1_{(U_{Y_1} \le p)}, \ldots , 1_{(U_{Y_T} \le p)} \right)$, and for the treatment group, $(X_1, \ldots , X_S, Y_1, \ldots , Y_T)= \left( 1_{(U_{X_1} \le p)}, \ldots , 1_{(U_{X_S} \le p)}, 1_{(U_{Y_1} \le p^\prime )}, \ldots , 1_{(U_{Y_T} \le p^\prime )} \right)$. Two GEE logistic regression models are considered as follows.

$$\begin{aligned} \text {GEE Model 1:} \quad \log \left( \frac{P(Y_{ijt}=1)}{1-P(Y_{ijt}=1)} \right)= & {} \beta _0+ \beta _1 \text {Treat}_{ij} + \beta _2 X_{ij+}, \\ \text {GEE Model 2:} \quad \log \left( \frac{P(Y_{ijt}=1)}{1-P(Y_{ijt}=1)} \right)= & {} \beta _0+ \beta _1 \text {Treat}_{ij}+ \beta _2 X_{\text {log}, ij+} , \end{aligned}$$

where $Y_{ijt}$ is the multiple binary outcome, $t=1,\ldots , T$. The treatment indicator $\text {Treat}_{ij}=0$ for placebo and 1 for treatment, $X_{ij+}=X_{ij 1}+ \cdots + X_{ij S}$ and $X_{\text {log}, ij+}=\text {log} \left[ (X_{ij+}+1/2)/(2-X_{ij+}+1/2) \right] , i=0, 1,\ j=1,\ldots , n_{i}$.

Consider $H_0: \beta _1=0$ vs. $H_1: \beta _1\ne 0$. Similarly, assume $p=0.4, p^\prime =\frac{p e^{\beta _1}}{1-p+p e^{\beta _1}}$ and $n_0=n_1=\{50, 100, 150\}$. Different $\rho _{XY}$ and $\rho _X=\rho _Y$ are considered as $\rho _{XY}=0.5, \rho _X=\rho _Y=\{0.6, 0.7, 0.8, 0.9\}$; $\rho _{XY}=0.6, \rho _X=\rho _Y=\{0.7, 0.8, 0.9\}$ and $\rho _{XY}=0.7, \rho _X=\rho _Y=\{0.8, 0.9\}$. We conduct simulation studies with treatment effect coefficient $\beta _1=0$ to obtain Type I error probability and $\beta _1=0.5$ to obtain power. We compare the power under 9 different scenarios of $S=\{1, \ldots , 9\}$ and $T=10-S$, then find $S_{\text {opt}}$ that has the highest power. For $T=1$, we use logistic regression. For other scenarios, we use GEE logistic regression. Again, to avoid perfect separation for small samples, we only conduct the simulation studies using GEE Model 2 when $n_0=n_1=50$ .

During the simulation studies, we found that the Type I error probabilities for GEE logistic regression ($T \ge 2$) are hard to control. This is because when the sample size is small, the robust sandwich estimator is biased downward for estimating $\text {var}(\hat{\beta }_1)$ [16, 17] and the *Z*-statistics $\hat{\beta }_1 / \sqrt{\text {var}(\hat{\beta }_1)}$ would be overestimated and then increase the Type I error probabilities. That will make the power comparison between $T=1$ (logistic regression) and $T \ge 2$ (GEE) to be inaccurate. Hence, the empirical calibration of the *Z*-test is applied to control the Type I error probabilities of GEE, and we obtain the empirical power for comparison.

We first obtain the *Z*-statistics $\hat{\beta }_1 / \sqrt{\text {var}(\hat{\beta }_1)}$ under $H_0$, which follows *N*(0, 1) when $n \rightarrow \infty$. But since our sample size is not infinity, the $(\alpha /2)\times 100\%$ and $(1-\alpha /2)\times 100\%$ quantiles of the *Z*-statistics are not the quantiles of *N*(0, 1). To calibrate the Type I error probabilities at level $\alpha$, we obtain the empirical $(\alpha /2)\times 100\%$ and $(1-\alpha /2)\times 100\%$ quantiles of the *Z*-statistics from simulation studies. By definition, those empirical quantiles have Type I error probabilities exactly equal to $\alpha$. We then use these empirical quantiles to calibrate the power. Similar ideas of using p-value empirical calibration to control the Type I error probabilities are discussed by several authors [18, 19]. To make it consistent, we calibrate the Type I error probabilities at level $\alpha$ for not only the GEE regression ($T \ge 2$) but also the logistic regression ($T=1$), then compare the calibrated power for different $S=\{1, \ldots , 9\}$.

The original Type I error probabilities (without calibration) of multiple binary outcomes using GEE models are shown in Tables S2-S4. The upper bound of 95% confidence interval for estimating the Type I error probability at $\alpha = 0.05$ is $0.05+1.96 \times \sqrt{(0.05)(0.95)/20000}=0.053$. The inflated original Type I error probabilities ($>0.053$) are shown in italic font in these tables. When $n_0=n_1=50$, the original observed Type I error probabilities are hard to control under the GEE logistic regression (Table S2). With a larger sample size ($n_0=n_1=100, 150$), more observed Type I error probabilities can be controlled (Tables S3, S4). The calibrated Type I error probabilities are all equal to $\alpha =0.05$ (not shown in the tables).

**Fig. 2:**
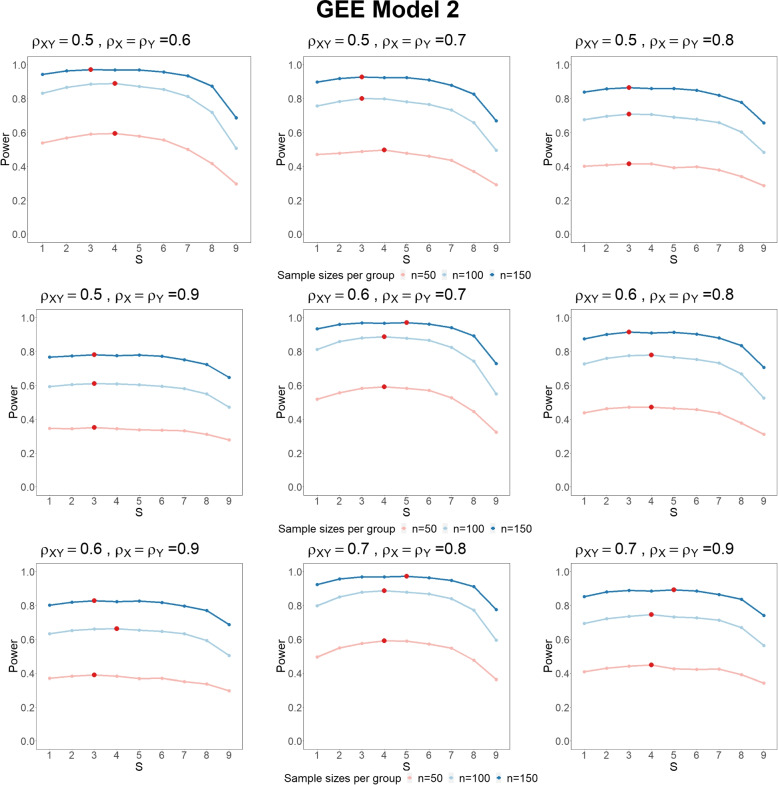
The calibrated power of multiple binary outcomes using GEE Model 2, with total number of visits $M=10$, sample size per group $n=n_0=n_1=\{50, 100, 150\}$ under different $\rho _{XY}, \rho _X$ and $\rho _Y$. The number of pre-treatment measurements $S=\{1, \ldots , 9\}$. The optimal number of pre-treatment visits $S_{\text {opt}}$ are highlighted in red points

The calibrated power comparison for $S=\{1, \ldots , 9\}$ using two GEE logistic regression models are shown in Figures 2 and S1. The power curves first increase from $S=1$ to $S=3$. For $3< S \le M/2$, there is little change in power. When $S > M/2$, the power curves decrease to a minimum at $S=M-1$. The optimal number of pre-treatment visits $S_{\text {opt}}$ are highlighted in red, showing that $S_{\text {opt}}$ are less than or equal to $M/2=5$. Hence, when $M = 10$, repeating pre-treatment measurements with $2< S \le 5$ would provide the optimal power. The optimal pre-post allocations in GEE logistic regressions have similar conclusions as the linear models, that is, $S_{\text {opt}} < M/2$ when $\rho _X=\rho _Y$. Measurements taken after the randomization can be more informative since we treat the pre-treatment measurements as covariates.

Overall, the results for the multiple binary outcomes with GEE logistic regression are similar to those for the continuous outcomes with the ANCOVA model. The proposed method extends well to the non-linear models through Monte Carlo simulation studies. The closed-form formulas for sample size, power, and $S_{\text {opt}}$ calculations under non-linear models require future investigations.

## Discussion

In this article, we demonstrate the merits of having multiple pre-treatment measurements for both continuous and discrete responses in pre-post designs. We consider the sample size calculation for the ANCOVA model when the pre-treatment measures are included as covariates under a general correlation structure. Then we propose an optimal design under a specific constraint that the total number of pre-treatment and post-treatment visits is fixed. Simulation studies were conducted for binary outcomes, suggesting that the insights from the linear model extend well to GEE logistic regression.

The prior information on the correlation structure is required to determine sample size and the optimal pre-post allocation. Designers can obtain the prior information of correlation structure based on some examples of clinical trials (e.g., Table III in [5]). Besides, an adaptive design can be further considered to estimate those correlations during the interim analysis. One can start the design with prior information based on other examples of clinical trials. During the interim analysis, one can use Stage 1 data to estimate the correlation structure, then adapt the sample size formula and the pre-post allocation for Stage 2.

Extensions of the ANCOVA model include the considerations of different time intervals between measurements and alternative correlation structures such as an autoregressive structure:
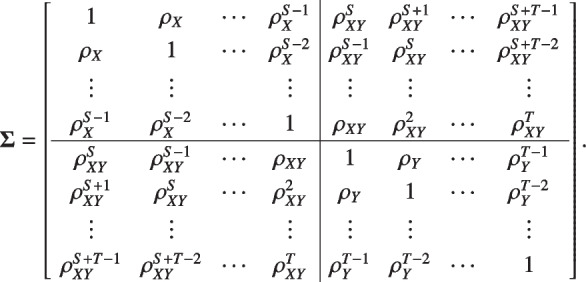


In clinical trial designs, the time intervals of pre-treatment visits and post-treatment visits could be equally spaced. However, if the time interval between the visits increases, the correlation tends to decline [5]. When the time intervals between visits are not equally spaced, one can consider an autoregressive structure or a more general correlation structure that assumes the correlations between all pairs of measurements are different. We leave this as future work for more thorough investigations. Like many other statistical methods, the proposed ANCOVA model could also be extended to adjust for covariates other than the baseline measurement of the outcome and further improve precision [20]. Similar to the idea of measuring the pre-treatment outcome multiple times, collecting other covariates multiple times may help further improve the framework. However, one needs to carefully address the potential correlation between the key covariate in ANCOVA (e.g. average baseline scores) and other covariates. Another possible extension is in observational studies. Though our method is proposed under the framework of classic clinical trials, it shares some similarities with the Difference-in-Difference (DID) technique, which is a quasi-experimental design applied in observational settings where exchangeability cannot be assumed between the treatment and control groups. Though DID is a technique to remove biases in the post-intervention period after data collection, how to adapt our method to this scenario and obtain the optimal pre-post allocation before the data collection could be a future research topic.

There are still remaining questions to be discussed. Several authors, including Liang and Zeger [15] and Tango [10], have recommended analyzing the pre-treatment measurements as additional outcomes through mixed effect models rather than treating them as covariates. Comparison between using a single baseline as a covariate or dependent variable were discussed by Liu et al. [21] and Wan [22]. It would be interesting to compare the repeating baselines sample size calculation between the ANCOVA model and the linear mixed effect model, then consider the optimal pre-post allocation of linear and logistic mixed effect model for both continuous and binary outcomes. It is noteworthy that the ANCOVA model might be misspecified for the discrete outcomes. Extension to discrete responses with non-linear models can be a future direction to deal with this issue. Regarding non-linear models, it would be helpful to strengthen the theoretical analysis for logistic mixed-effect models by simulation studies or closed-form formulations.

Another future direction is the three-arm clinical trial, which includes an experimental treatment, an active reference treatment, and a placebo group [23–25]. Besides, one can further consider, given a constraint of the total cost, how to obtain the optimal choice of sample size and the number of pre-treatment and post-treatment visits to maximize the power function. Generally speaking, if the costs of each pre-post visit are high, one can tend to select a larger sample size. In contrast, if the expense of recruiting each patient is high, then we would expect to get a smaller sample size but repeat more pre-treatment and post-treatment measurements.

Although using both screening and baseline can be more powerful than using a single baseline, sometimes there are ethical concerns about having multiple pre-treatment visits in clinical trials. For trials and diseases that require treatment immediately after the baseline visit, it could be impractical and unethical to repeat the pre-treatment measurements [5]. Finally, a potential benefit of repeating pre-post measurements is to reduce the impact of missing values in the ANCOVA analysis, especially for missing baseline data. This also merits further discussion.

## Conclusion

We address the advantages of using multiple pre-treatment and post-treatment measurements in randomized clinical trials. For the ANCOVA model, the sample size formula under general correlation structures is considered, and we derive the optimal number of pre/post measurements given the total number of visits. Repetition of the follow-up measurements is generally more beneficial than repeating the baselines, but the latter can provide nonnegligible improvement of the efficiency in repeated measures designs. Simulation studies are conducted for binary measurements, which have similar conclusions as for the linear model.

## Supplementary Information

**Additional file 1.**

## Data Availability

All R codes are available at https://doi.org/10.5281/zenodo.7594938 [26].

## Abbreviations

ANCOVA: Analysis of covariance
GEE: Generalized estimating equations
