# Supplementary material for: The optimal pre-post allocation for randomized clinical trials
Source: BMC Med Res Methodol. 2023 Mar 28;23:72. doi: 10.1186/s12874-023-01893-w (PMC10045175; doi:10.1186/s12874-023-01893-w)
Supplement: Supplementary file 1 — Additional file 1. [file 12874_2023_1893_MOESM1_ESM.pdf]

Supplemental Material

Table S1: **The Type I error probabilities of multiple continuous outcomes using ANCOVA model**, with total number of visits $M = 10$, sample size $n_0 = n_1 = \{50, 100, 150\}$ under different ρ_{XY}, ρ_X and ρ_Y. The number of pre-treatment measurements $S = \{1, \ldots, 9\}$.

	$S = 1$	$S = 2$	$S = 3$	$S = 4$	$S = 5$	$S = 6$	$S = 7$	$S = 8$	$S = 9$
	$\rho_{XY} = 0.5, \rho_X = \rho_Y = 0.6$								
$n_0 = n_1 = 50$	0.0518	0.0506	0.0512	0.0504	0.0482	0.05	0.0496	0.0515	0.0494
$n_0 = n_1 = 100$	0.0494	0.0517	0.0493	0.0482	0.0498	0.0512	0.0528	0.05	0.0511
$n_0 = n_1 = 150$	0.0517	0.0504	0.0489	0.0522	0.0507	0.0486	0.0498	0.0491	0.0505
	$\rho_{XY} = 0.5, \rho_X = \rho_Y = 0.7$								
$n_0 = n_1 = 50$	0.0515	0.0506	0.0507	0.0498	0.0483	0.0502	0.0496	0.0511	0.0492
$n_0 = n_1 = 100$	0.0488	0.0511	0.0495	0.0486	0.0503	0.0512	0.0528	0.0503	0.0508
$n_0 = n_1 = 150$	0.052	0.0501	0.0484	0.0532	0.0508	0.0484	0.0502	0.0479	0.051
	$\rho_{XY} = 0.5, \rho_X = \rho_Y = 0.8$								
$n_0 = n_1 = 50$	0.0518	0.0502	0.0512	0.0497	0.0486	0.0504	0.0498	0.0511	0.0496
$n_0 = n_1 = 100$	0.0486	0.0512	0.049	0.0482	0.0491	0.0513	0.0532	0.0503	0.0506
$n_0 = n_1 = 150$	0.0516	0.0501	0.0482	0.0529	0.0512	0.0484	0.0498	0.0481	0.0514
	$\rho_{XY} = 0.5, \rho_X = \rho_Y = 0.9$								
$n_0 = n_1 = 50$	0.052	0.0498	0.0514	0.0493	0.0484	0.0502	0.0503	0.0513	0.0496
$n_0 = n_1 = 100$	0.0482	0.0511	0.0481	0.0482	0.0491	0.051	0.0522	0.0506	0.0507
$n_0 = n_1 = 150$	0.0518	0.05	0.0478	0.0528	0.0504	0.0486	0.0497	0.0482	0.0512
	$\rho_{XY} = 0.6, \rho_X = \rho_Y = 0.7$								
$n_0 = n_1 = 50$	0.0496	0.0516	0.0519	0.0503	0.0488	0.05	0.0496	0.0516	0.0496
$n_0 = n_1 = 100$	0.049	0.0516	0.0499	0.049	0.0497	0.0508	0.0528	0.0499	0.0512
$n_0 = n_1 = 150$	0.0515	0.0506	0.0489	0.0526	0.0512	0.0478	0.0502	0.0488	0.0505
	$\rho_{XY} = 0.6, \rho_X = \rho_Y = 0.8$								
$n_0 = n_1 = 50$	0.0504	0.0508	0.0512	0.0503	0.0483	0.0503	0.0495	0.0516	0.0494
$n_0 = n_1 = 100$	0.0494	0.0516	0.0493	0.0482	0.0503	0.0513	0.0528	0.0502	0.0511
$n_0 = n_1 = 150$	0.0516	0.0504	0.0488	0.052	0.0508	0.0485	0.05	0.0488	0.0506
	$\rho_{XY} = 0.6, \rho_X = \rho_Y = 0.9$								
$n_0 = n_1 = 50$	0.0514	0.0509	0.0507	0.0497	0.0483	0.0502	0.0496	0.0512	0.0492
$n_0 = n_1 = 100$	0.0494	0.0516	0.0496	0.0486	0.05	0.0512	0.053	0.0504	0.0508
$n_0 = n_1 = 150$	0.0517	0.0506	0.0484	0.0532	0.051	0.0483	0.0502	0.048	0.051
	$\rho_{XY} = 0.7, \rho_X = \rho_Y = 0.8$								
$n_0 = n_1 = 50$	0.0508	0.051	0.052	0.0499	0.0488	0.05	0.0497	0.0513	0.0493
$n_0 = n_1 = 100$	0.0483	0.0515	0.0502	0.0492	0.0498	0.0508	0.0528	0.0498	0.0509
$n_0 = n_1 = 150$	0.0518	0.0505	0.0495	0.0523	0.0501	0.048	0.0505	0.0485	0.0505
	$\rho_{XY} = 0.7, \rho_X = \rho_Y = 0.9$								
$n_0 = n_1 = 50$	0.0506	0.0516	0.0518	0.0502	0.0486	0.0502	0.0494	0.0517	0.0493
$n_0 = n_1 = 100$	0.0485	0.0517	0.0498	0.0487	0.0495	0.0512	0.0528	0.0498	0.0512
$n_0 = n_1 = 150$	0.0516	0.0508	0.0488	0.0528	0.0512	0.0483	0.0498	0.0489	0.0505

Table S2: **The original Type I error probabilities of multiple binary outcomes using GEE Model 2**, with total number of visits $M = 10$, sample size $n_0 = n_1 = 50$ under different ρ_{XY}, ρ_X and ρ_Y. The number of pre-treatment measurements $S = \{1, \ldots, 9\}$. The inflated Type I error probabilities are shown in italic font. The calibrated type I error probabilities all equal to $\alpha = 0.05$ (not shown in the table).

$S = 1$	$S = 2$	$S = 3$	$S = 4$	$S = 5$	$S = 6$	$S = 7$	$S = 8$	$S = 9$
$\rho_{XY} = 0.5, \rho_X = \rho_Y = 0.6$								
0.054	*0.0556*	*0.0544*	*0.0566*	*0.0549*	*0.054*	*0.0559*	0.0523	0.048
$\rho_{XY} = 0.5, \rho_X = \rho_Y = 0.7$								
0.0524	*0.0544*	*0.0554*	0.0522	*0.0552*	*0.0534*	*0.0544*	*0.0544*	0.0477
$\rho_{XY} = 0.5, \rho_X = \rho_Y = 0.8$								
0.0538	*0.0536*	0.052	0.0529	*0.0535*	0.0512	*0.0538*	0.0513	0.0488
$\rho_{XY} = 0.5, \rho_X = \rho_Y = 0.9$								
0.0528	0.0524	0.0518	0.0522	0.0508	0.0511	*0.0532*	0.0506	0.0482
$\rho_{XY} = 0.6, \rho_X = \rho_Y = 0.7$								
0.0561	*0.0556*	*0.0548*	*0.0561*	*0.0566*	*0.0551*	*0.0554*	0.0516	0.0475
$\rho_{XY} = 0.6, \rho_X = \rho_Y = 0.8$								
0.0549	*0.0549*	*0.0546*	*0.0544*	*0.0531*	0.0526	*0.0541*	0.051	0.048
$\rho_{XY} = 0.6, \rho_X = \rho_Y = 0.9$								
0.0546	0.0516	0.0527	0.0527	0.052	0.0517	*0.054*	0.049	0.048
$\rho_{XY} = 0.7, \rho_X = \rho_Y = 0.8$								
0.0563	*0.0567*	*0.0551*	*0.0552*	*0.0539*	*0.0554*	*0.0535*	0.0512	0.0457
$\rho_{XY} = 0.7, \rho_X = \rho_Y = 0.9$								
0.055	*0.0534*	0.0529	0.0521	0.0522	0.0528	0.0516	0.0488	0.0443

Table S3: **The original Type I error probabilities of multiple binary outcomes using GEE Models 1 and 2**, with total number of visits $M = 10$, sample size $n_0 = n_1 = 100$ under different ρ_{XY}, ρ_X and ρ_Y. The number of pre-treatment measurements $S = \{1, \ldots, 9\}$. The inflated Type I error probabilities are shown in italic font. The calibrated type I error probabilities all equal to $\alpha = 0.05$ (not shown in the table).

	$S = 1$	$S = 2$	$S = 3$	$S = 4$	$S = 5$	$S = 6$	$S = 7$	$S = 8$	$S = 9$
	$\rho_{XY} = 0.5, \rho_X = \rho_Y = 0.6$								
GEE Model 1	0.0526	0.0524	0.0524	0.0511	*0.0532*	*0.0535*	0.0518	0.0506	0.0508
GEE Model 2	0.0526	0.0524	0.0522	0.0511	0.0528	*0.0541*	0.0527	0.0507	0.0502
	$\rho_{XY} = 0.5, \rho_X = \rho_Y = 0.7$								
GEE Model 1	0.0523	*0.0545*	0.0514	0.0515	0.0522	*0.0534*	0.0522	0.0512	0.0516
GEE Model 2	0.0523	*0.0545*	0.0517	0.0511	0.0517	*0.0535*	0.0528	0.0514	0.0504
	$\rho_{XY} = 0.5, \rho_X = \rho_Y = 0.8$								
GEE Model 1	0.0512	0.0524	0.0529	0.0508	0.0512	0.0528	*0.0541*	0.0506	0.0524
GEE Model 2	0.0512	0.0524	0.0526	0.0508	0.0505	0.0524	*0.0532*	0.05	0.0521
	$\rho_{XY} = 0.5, \rho_X = \rho_Y = 0.9$								
GEE Model 1	0.0492	0.0502	0.0522	0.0494	0.0501	0.0528	*0.0534*	0.051	0.0525
GEE Model 2	0.0492	0.0502	0.0515	0.0491	0.0498	*0.0531*	*0.0532*	0.0515	0.0518
	$\rho_{XY} = 0.6, \rho_X = \rho_Y = 0.7$								
GEE Model 1	0.0528	*0.0532*	0.0512	0.0527	0.0512	0.0528	0.0511	0.0504	0.0498
GEE Model 2	0.0528	*0.0532*	0.0506	0.0528	0.0512	0.0528	0.0517	0.0498	0.0512
	$\rho_{XY} = 0.6, \rho_X = \rho_Y = 0.8$								
GEE Model 1	0.0509	*0.0541*	0.0515	0.0502	0.052	0.0528	*0.0552*	0.0497	0.051
GEE Model 2	0.0509	*0.0541*	0.0517	0.0498	0.0526	*0.0532*	*0.0556*	0.0499	0.0514
	$\rho_{XY} = 0.6, \rho_X = \rho_Y = 0.9$								
GEE Model 1	0.0497	0.0492	0.0512	0.0504	0.0505	0.0524	0.0521	0.0503	0.0501
GEE Model 2	0.0497	0.0492	0.0516	0.05	0.0505	*0.0536*	0.0522	0.0501	0.05
	$\rho_{XY} = 0.7, \rho_X = \rho_Y = 0.8$								
GEE Model 1	0.0509	0.053	*0.0532*	0.051	0.051	*0.0539*	0.0514	0.0504	0.0527
GEE Model 2	0.0509	0.053	0.0527	0.0511	0.0505	*0.0534*	0.0514	0.0499	0.0524
	$\rho_{XY} = 0.7, \rho_X = \rho_Y = 0.9$								
GEE Model 1	0.0503	0.0528	0.051	0.051	0.0494	0.0523	0.0527	0.0516	0.0518
GEE Model 2	0.0503	0.0528	0.0504	0.0502	0.0504	0.0526	*0.0539*	0.0508	0.0512

Table S4: **The original Type I error probabilities of multiple binary outcomes using GEE Models 1 and 2**, with total number of visits $M = 10$, sample size $n_0 = n_1 = 150$ under different ρ_{XY}, ρ_X and ρ_Y. The number of pre-treatment measurements $S = \{1, \ldots, 9\}$. The inflated Type I error probabilities are shown in italic font. The calibrated type I error probabilities all equal to $\alpha = 0.05$ (not shown in the table).

	$S = 1$	$S = 2$	$S = 3$	$S = 4$	$S = 5$	$S = 6$	$S = 7$	$S = 8$	$S = 9$
	$\rho_{XY} = 0.5, \rho_X = \rho_Y = 0.6$								
GEE Model 1	0.053	*0.0558*	0.0488	0.052	0.0516	0.0496	0.0508	0.0459	0.051
GEE Model 2	0.053	*0.0558*	0.0495	0.0522	0.0517	0.0493	0.0508	0.0457	0.0503
	$\rho_{XY} = 0.5, \rho_X = \rho_Y = 0.7$								
GEE Model 1	0.0522	*0.0532*	0.0515	0.0501	0.0503	0.0488	0.051	0.045	0.051
GEE Model 2	0.0522	*0.0532*	0.051	0.0509	0.05	0.0482	0.0508	0.0449	0.0505
	$\rho_{XY} = 0.5, \rho_X = \rho_Y = 0.8$								
GEE Model 1	0.053	*0.0549*	0.0513	0.051	0.0512	0.0481	0.0518	0.0454	0.0505
GEE Model 2	0.053	*0.0549*	0.0516	0.0503	0.0513	0.0484	0.0526	0.0452	0.0495
	$\rho_{XY} = 0.5, \rho_X = \rho_Y = 0.9$								
GEE Model 1	0.052	*0.0534*	0.0492	0.0486	0.0516	0.0482	0.0522	0.0463	0.05
GEE Model 2	0.052	*0.0534*	0.0493	0.0488	0.0515	0.0486	0.0528	0.0463	0.0502
	$\rho_{XY} = 0.6, \rho_X = \rho_Y = 0.7$								
GEE Model 1	*0.0534*	*0.055*	0.0493	0.0502	0.05	0.0499	0.0529	0.0471	0.0497
GEE Model 2	*0.0534*	*0.055*	0.0492	0.051	0.0508	0.05	*0.0534*	0.0462	0.0492
	$\rho_{XY} = 0.6, \rho_X = \rho_Y = 0.8$								
GEE Model 1	*0.0539*	*0.0541*	0.0506	0.0494	0.0502	0.0491	0.0516	0.046	0.0502
GEE Model 2	*0.0539*	*0.0541*	0.0508	0.05	0.0504	0.0491	0.0527	0.0458	0.0501
	$\rho_{XY} = 0.6, \rho_X = \rho_Y = 0.9$								
GEE Model 1	0.0524	*0.0536*	0.0503	0.0494	0.0515	0.0493	0.0513	0.0454	0.0486
GEE Model 2	0.0524	*0.0536*	0.0508	0.0493	0.052	0.0498	0.0514	0.0454	0.0486
	$\rho_{XY} = 0.7, \rho_X = \rho_Y = 0.8$								
GEE Model 1	*0.0551*	*0.0556*	0.051	0.0491	0.0502	0.0488	0.0517	0.0458	0.0504
GEE Model 2	*0.0551*	*0.0556*	0.0505	0.0488	0.0513	0.0491	0.0518	0.0456	0.0497
	$\rho_{XY} = 0.7, \rho_X = \rho_Y = 0.9$								
GEE Model 1	*0.0532*	*0.0538*	0.0492	0.0512	0.0524	0.0494	0.0516	0.0465	0.0495
GEE Model 2	*0.0532*	*0.0538*	0.0493	0.0512	0.0519	0.0492	0.0527	0.047	0.0486

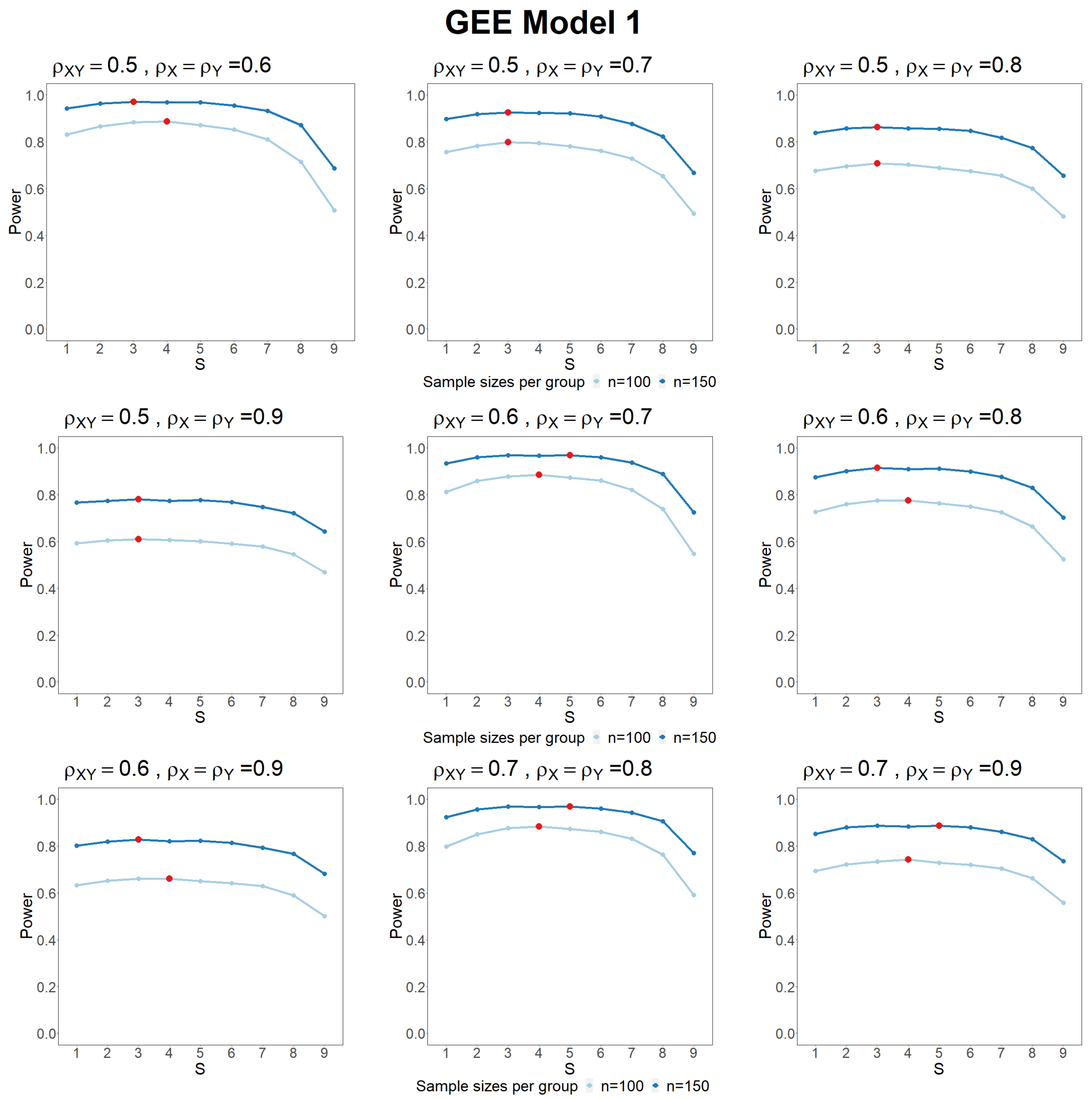

Figure S1: **The calibrated power of multiple binary outcomes using GEE Model 1**, with total number of visits $M = 10$, sample size per group $n = n_0 = n_1 = \{100, 150\}$ under different ρ_{XY}, ρ_X and ρ_Y. The number of pre-treatment measurements $S = \{1, \ldots, 9\}$. The optimal number of pre-treatment visits S_{opt} are highlighted in red points.
